# Novelty, Challenge, and Practice: The Impact of Intensive Language Learning on Attentional Functions

**DOI:** 10.1371/journal.pone.0153485

**Published:** 2016-04-27

**Authors:** Thomas H. Bak, Madeleine R. Long, Mariana Vega-Mendoza, Antonella Sorace

**Affiliations:** 1 Department of Psychology, School of Philosophy, Psychology and Language Sciences, University of Edinburgh, Edinburgh, United Kingdom; 2 Centre for Cognitive Ageing and Cognitive Epidemiology (CCACE), Edinburgh, United Kingdom; 3 Department of Linguistics, School of Philosophy, Psychology and Language Sciences, University of Edinburgh, Edinburgh, United Kingdom; University of Akron, UNITED STATES

## Abstract

We investigated the impact of a short intensive language course on attentional functions.

We examined 33 participants of a one-week Scottish Gaelic course and compared them to 34 controls: 16 active controls who participated in courses of comparable duration and intensity but not involving foreign language learning and 18 passive controls who followed their usual routines. Participants completed auditory tests of attentional inhibition and switching. There was no difference between the groups in any measures at the beginning of the course. At the end of the course, a significant improvement in attention switching was observed in the language group (*p* < .001) but not the control group (*p* = .127), independent of the age of participants (18–78 years). Half of the language participants (n = 17) were retested nine months after their course. All those who practiced Gaelic 5 hours or more per week improved from their baseline performance. In contrast, those who practiced 4 hours or fewer showed an inconsistent pattern: some improved while others stayed the same or deteriorated. Our results suggest that even a short period of intensive language learning can modulate attentional functions and that all age groups can benefit from this effect. Moreover, these short-term effects can be maintained through continuous practice.

## 1. Introduction

Few topics have recently generated as much controversy as the question of possible cognitive benefits associated with bilingualism, particularly in areas such as executive functions and attention. The evidence is inconsistent. Some studies show better results in bilinguals, from childhood [1] to old age [2] and dementia [3]; others find no difference [4]. However, as documented by de Bruin et al [5], very few studies show an opposite effect, namely a bilingual disadvantage.

Most studies thus far have examined “classic” bilingualism: early acquisition and balanced command of different languages. However, recent research into people who have learned languages in adulthood and without reaching native-like proficiency suggests similar cognitive effects as in the classic bilinguals [6–9]. These findings open a new set of questions: How much language learning is necessary before the first cognitive changes become detectable? How much practice is needed to sustain them? Do they occur in people of all ages, even in the elderly?

In this study, we set out to determine whether learning a new language would lead to an improvement in cognitive performance as early as one week after an intensive course. We examined learners of Scottish Gaelic: a Celtic language different from English in phonology, vocabulary, and word order, with a complex grammar and unfamiliar spelling [10] posing considerable challenges to its learners. The control group consisted of two subgroups: *active controls* who participated in courses of comparable duration and intensity but not involving foreign language learning and *passive controls* who followed their usual routines.

To assess cognitive functions we used subtests from the Test of Everyday Attention [11], measuring attentional inhibition and switching, functions which play a central role in the current understanding of cognitive processing in bilinguals [12, 13]. In a recent study using these tests [14], fourth year language (but not literature) students outperformed their first year counterparts, suggesting a positive effect of intensive language learning on attentional performance. However, while Vega-Mendoza et al examined different students at two different stages of their academic career, the present study investigates potential differences *within* the same participants.

We predicted that all three groups would be indistinguishable from each other in their baseline performance. Since the parallel versions of the Test of Everyday Attention [11] were designed to avoid practice effects, so that the test can be used in longitudinal studies and in monitoring the effects of neuro-rehabilitation, we did not expect to find any changes in performance between the first and the second assessment in the passive control group. In contrast, given the growing evidence for beneficial cognitive effects of different types of mental exercise [15, 16], we predicted that both the active controls and the language group would improve after one week of their respective intensive courses. In view of the particular challenges associated with learning an unfamiliar language, we hypothesized that the improvement would be more pronounced in language learners [17]). Finally, we speculated that long-term language practice could help to maintain cognitive improvement.

## 2. Methodology

### 2.1. Participants

A total of 76 volunteers participated in the study: 36 language learners and 40 controls. Language learners were recruited from Sabhal Mòr Ostaig, a Gaelic college on the Isle of Skye, Scotland, and were tested before and after a one-week intensive Gaelic course. The testing was conducted over three weeks in Summer 2014. Everyone enrolled in language courses was invited via email to participate in the study. All those who agreed were tested. The students had an average of 14 hours of language classes between the first and second testing and were offered Gaelic entertainment in the evening (e.g. concerts, films, conversation circle).

The control group consisted of 40 individuals not enrolled in a language course. Recruitment was comparable to that of the language learners in that the controls received a written invitation and all those who signed up were tested. The group was further subdivided into active and passive controls to examine whether any potential cognitive changes in language learning were course-specific or due to general stimulation in an intensive course environment. Active controls (n = 16) were enrolled in intensive courses with a similar schedule to those of the Gaelic students, but were not learning an unfamiliar language. The courses included CELTA training (an English language teaching qualification for those fluent in English) at the Randolph School of English (*m* = 19.5 hours between testing), art courses at Leith School of Art (*m* = 12 hours between testing), and a documentary film course offered by the University of Edinburgh (*m* = 15 hours between testing). Both language and non-language courses were taught by multiple tutors; hence, it is unlikely that potential differences might be due to the personality and/or teaching style of individual tutors. Passive controls (n = 24) were recruited through the University of Edinburgh Psychology Volunteer Panel, were not enrolled in any type of intensive course at the time of testing, and were following their usual daily routine.

Two volunteers (one each from the language and passive control groups) withdrew during the first session and their data were removed. In order to match the groups by age, gender, and education, two language participants and one passive control participant were excluded because of level of education (secondary school degree only) and four passive control participants were excluded because of age (80 years or above). To make sure that these exclusions did not influence the overall results, the comparison of the Elevator Task, Elevator Task with Distraction, and Elevator Task with Reversal (as reported in the results section) was conducted twice: with and without the excluded participants. The results were practically the same, with the differences smaller than 1.5%; we subsequently report the results of the matched groups.

Within the matched groups, there were no significant differences between the language group (n = 33) and control group (n = 34) in age: language group: 50.06 ±18.28, control group: 48.24 ±16.37; gender (percentage females): language group: 60.60%, control group: 76.47% or education (percentage with degree): language group: 93.93%, control group: 88.23%. Likewise, no significant differences were found in the same variables between the active (n = 16) and passive (n = 18) control group—age: active controls: 43.69 ± 17.68, passive controls: 52.28 ±14.42; gender (percentage females): active controls: 81.25%, passive controls: 72.22%; education (percentage with degree): active controls: 93.75%, passive controls: 83.33%.

In addition, all groups completed a comprehensive language background questionnaire to assess knowledge of foreign languages (see S1 Appendix). This self-evaluation separates an individual’s command of all languages of which he/she has at least basic knowledge into four domains: expression, comprehension, reading, and writing. Each domain is then rated using a 5 point scale (from 1 = basic to 5 = fluent) and the composite score of all known languages (including knowledge of Gaelic before the beginning of the course) are calculated for each individual. This composite score was not different between the language and the control group (language group: 37.36 ± 15.04, control group: 34.21 ± 13.35) or between the active and passive controls (active controls: 34.81 ± 13.66, passive controls: 33.67 ± 13.44).

### 2.2. Materials/Procedures

The Test of Everyday Attention [11] is a well-established clinical test, measuring different aspects of attention. It has been designed to diagnose subtle attentional deficits and monitor effects of neuro-rehabilitation in patients with different types of brain damage. For this reason, it includes three different versions of each test to prevent practice effects. More recently, the Test of Everyday Attention subtests—the Elevator Task, Elevator Task with Distraction, and Elevator Task with Reversal—have been successfully applied to examine the influence of early and late bilingualism [7] and foreign language learning [14] on attentional functions in young adults. The three subtests together take ca. 20 minutes and can be easily administered outside of laboratory settings, making them well suited for the field work involved in this study.

Each of the subtests were designed to measure distinct attentional components (sustained attention, selective attention, and attentional switching), requiring a separate assessment for each of these functions. Due to inherent task differences, performance can differ across tasks, as has been previously demonstrated [7]. For this reason, we did not calculate a composite score for the separate subtests, but rather analyzed each subtest separately.

*Elevator Task (auditory sustained attention*): Participants are asked to count tones of the same pitch presented at irregular intervals (n trials = 7).

*Elevator Task with Distraction (auditory selective attention/inhibition*): Participants are asked to count low tones, while ignoring interspersed high tones (n trials = 10).

*Elevator Task with Reversal (auditory attentional switching*): Participants are presented with high, middle, and low tones. The middle tones are to be counted while the high and low tones indicate whether to add or subtract middle tones (n trials = 10).

Results were measured in terms of accuracy of response. To avoid practice effects, a different version was given during each session, with the same versions and the same order (A, B, C) used in all three groups. Written informed consent was obtained from all participants prior to commencing the study. The study and the consent form were approved by the University of Edinburgh Psychology Ethics Committee.

### 2.3. Statistical analyses

Two-way mixed Analysis of Variances (ANOVAs) were conducted on the dependent variable of test score, with the within-subjects factor session (pre-learning, post-learning), and between-subject factor group (language, controls); t-tests were conducted to test whether the dependent variable of percentage improvement varied by group (language, controls). These tests were later repeated when comparing the subdivided active and passive controls, the different age groups, levels of Gaelic within the language group and the results of the follow-up assessment. In addition, a linear trend analysis was conducted when comparing passive and active controls with the language group. Bonferroni corrections were used where appropriate. Non-parametric tests were used in non-normally distributed data (Wilcoxon and U Mann-Whitney). All parametric and non-parametric analyses yielded the same pattern of results. Statistical analyses were performed using SPSS for Windows v.21. The data from the original study can be found in S2 Appendix and data from the follow-up can be found in S3 Appendix.

## 3. Results

### 3.1. Test of everyday attention subtests: language learners vs controls

#### 3.1.1. Elevator task

Both the language and control group scored close to ceiling on each session (Table 1). Accordingly, there was no main effect of session, *F*(1,65) = 1.26, *p* = .265, ηp2 = .019, group, *F*(1,65) = .128, *p* = .722, ηp2 = .002, or interaction, *F*(1,65) = .129, *p* = .720, ηp2 = .002.

**Table 1:** Summary of group performance on the Test of Everyday Attention subtests.

	Language Group	Controls		
	(n = 33)	Total (n = 34)	Active (n = 16)	Passive (n = 18)
**ET 1 Mean (SD)**	98.64 (4.38)	98.68 (4.32)	98.13 (5.12)	99.17 (3.54)
**ET 2 Mean (SD)**	99.09 (3.63)	99.56 (2.57)	99.06 (3.75)	100 (0)
**Improvement**	.45	.88	.93	.83
**ETD 1 Mean (SD)**	88.79 (22.61)	82.94 (25.17)	85.63 (21.90)	80.56 (28.17)
**ETD 2 Mean (SD)**	90.91 (20.06)	86.76 (22.12)	91.88 (9.81)	82.22 (28.61)
**Improvement**	2.12	3.82	6.25	1.66
**ETR 1 Mean (SD)**	59.7 (27.44)	57.06 (35.04)	60.63 (31.51)	53.89 (38.52)
**ETR 2 Mean (SD)**	78.48 (23.99)	62.94 (32.34)	73.75 (27.05)	53.33 (34.30)
**Improvement**	18.78	5.88	13.12	-.56

Mean, standard deviation, and improvement scores for all groups on the Test of Everyday Attention subtests.

Notes: ET: Elevator Task, ETD: Elevator Task with Distraction, ETR: Elevator Task with Reversal. Session is denoted by number (1 or 2).

#### 3.1.2. Elevator task with distraction

Similar to the first subtest, there was no main effect of session, *F*(1,65) = 2.54, *p* = .116, ηp2 = .038, group, *F*(1,65) = .948, *p* = .334, ηp2 = .014, or interaction *F*(1,65) = .208, *p* = .650, ηp2 = .003.

#### 3.1.3. Elevator task with reversal

The main effect of session was statistically significant, *F*(1,65) = 26.11, *p* < .001, ηp2 = .287, showing better performance overall in the second session. There was no main effect of group *F*(1,65) = 1.72, *p* = .195, ηp2 = .026, but the interaction between session and group was significant *F*(1,65) = 7.15, *p* = .009, ηp2 = .099. To follow up this interaction, pairwise comparisons were performed. The language group scored significantly better on the ETR from session 1 to session 2, *t* = 6.25, df = 32, *p* < .001, two-tailed, whereas the controls did not show any significant improvement over sessions, *t* = 1.57, df = 33, *p* = .127, two-tailed. Importantly, baseline performance did not differ across groups (language = 59.7, controls = 57.06, *p* = .733).

### 3.2. Linear trend analysis of improvement on the elevator task with reversal

**Fig 1:**
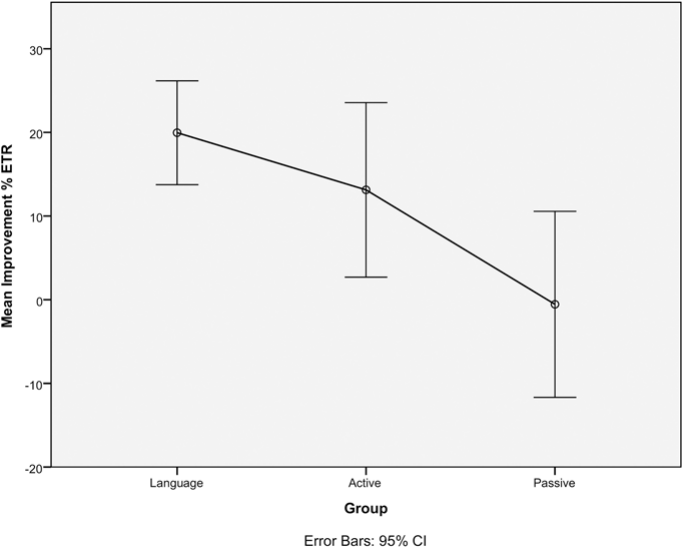
Linear trend performance on the Elevator Task with Reversal for the Language, Active, and Passive Control Groups.

In order to further examine the difference in improvement between session in the language, active, and passive control groups, the mean for each group in session 1 was subtracted from the mean for each group in session 2. A one-way independent measures ANOVA revealed an overall significant effect of improvement *F*(2,66) = 6.65, *p* = .002; follow up comparisons revealed that the language group was significantly different from the passive controls (*p* = .002). Although no differences were found between the language group and active controls and between the active controls and passive controls (*p* = .784 and .118, respectively), a significant linear trend *F*(1,64) = 12.87, *p* = .001 showed that proportionately the language group improved the most, followed by the active controls, and passive controls (Fig 1).

### 3.3. The influence of age on performance

In order to examine whether the rate of improvement on the Elevator Task with Reversal was dependent on the age of participants, the data for the language group’s performance was divided into three age groups of comparable size: 18–40 (n = 11), 41–60 (n = 9) and 61–78 (n = 13). The three age groups were not different in gender or education (*p =* .087 and *p* = .492, respectively). There was a statistically significant main effect of session (F(1,30) = 36.76, *p* < .001, ηp2 = .551), with a better performance in the second session). There was also a main effect of group (F(2,30) = 3.64, *p* = .038, ηp2 = .195). Post hoc tests with Bonferroni correction revealed that it was brought about by a significant difference in performance between the oldest and youngest groups (*p* = .035), with the youngest group scoring highest, and the oldest group lowest. The middle group took an intermediate position, with no significant differences to the youngest and the oldest group (all *p*’s> .05). There was no significant interaction (F(2,30) = .3, *p* = .740, ηp2 = .02).

The same analysis was carried out on the control group; participants were divided by age: 18–40 (n = 11), 41–60 (n = 15), and 61–80 (n = 8). No differences were found between the different age groups in gender *p* = .875 or education *p* = .240. Unlike the language learners, analysis revealed no main effect of session (F(1,31) = 3.42, *p* = .074, ηp2 = .099). There was a main effect of group (F(2,31) = 5, *p* = .013, ηp2 = .244) and follow-up tests (with Bonferroni correction) revealed that the oldest group had significantly lower scores than both the middle age group (*p* = .038) and the youngest group (*p* = .016); the middle and the youngest group did not differ significantly (*p* = 1.000). No significant interaction was found (F(2,31) = .947, p = .399, ηp2 = .058).

### 3.4. Difference within the language learning group: level of proficiency in Gaelic

To examine whether the rate of improvement was affected by previous knowledge of Gaelic, the language group’s performance on the Elevator Task with Reversal was divided by course level: complete beginners (Gaelic 1, n = 15), elementary (Gaelic 2, n = 8) and intermediate (Gaelic 3, n = 10) (Table 2). A t-test for age and chi-square tests for gender and level of education revealed no significant differences between the three groups (all *p*s > .2). The main effect of session was statistically significant (F(1,30) = 39.68, *p* < .001, ηp2 = .569), with better performance in the second session, but there was no main effect of group (F(1,30) = 1.29, *p* = .291, ηp2 = .079) or interaction (F(1,30) = 2.62, *p* = .089, ηp2 = .149).

**Table 2:** Elevator Task with Reversal performance for the three levels of Gaelic proficiency.

	Gaelic Level	Total (N)	ETR 1	ETR 2	Improvement
**ETR**	1	15	58.67 (28.25)	80.67 (21.2)	22
**Results**	2	8	66.25 (23.86)	91.25 (13.56)	25
	3	10	56 (30.62)	65 (29.16)	9

Notes: ETR: Elevator Task with Reversal. Session is denoted by number (1 or 2). () = SD.

### 3.5. Longitudinal follow-up

All of the language participants were contacted via email to participate in a follow-up study; 28 out of the 33 responded. Due to logistic reasons we were only able to retest those who lived in an accessible area of the UK, bringing the total number of the longitudinal follow-up group to 17. The participants were retested ca. 9 months after the course, with a repetition of the Test of Everyday Attention subtests and a questionnaire asking on average how many hours of Gaelic per week they practiced since the summer course. The reported hours ranged from 0 to 22.5/week with a median of 4/week. An exploratory inspection of the longitudinal follow-up data suggested the existence of a possible threshold between 4 and 5 hours of practice per week: in those who practiced more the performance on Session 3 was consistently better than the baseline (Table 3). Of those who practiced less, some improved, some deteriorated and some stayed the same.

**Table 3:** Individual performance on the Elevator Task with Reversal pre-course and nine months later.

	Post-Course Hrs/Wk of Gaelic Study	Performance ETR 1	Performance ETR 3	Improvement ETR 1 to ETR 3
	0	90	100	10
	0.5	40	10	-30
	1	70	100	30
	1	70	60	-10
	1	10	60	50
	1.5	40	30	-10
	3	10	10	0
**ETR**	4	80	90	10
**Results**	4	90	80	-10
	5	80	100	20
	5.5	40	70	30
	5.5	20	30	10
	5.5	40	60	20
	6	30	40	10
	10.5	70	90	20
	12.5	80	100	20
	22.5	80	100	20

Notes: ETR: Elevator Task with Reversal. Session is denoted by number (1 or 3).

As a first step, we examined whether those participants who practiced more were different from those who practiced less in terms of their demographic variables or baseline performance on the Elevator Task with Reversal. To do so, the participants were divided into those who practiced 4 hours or fewer (n = 9) versus 5 hours or more (n = 8). A t-test for age and chi-square tests for gender and level of education revealed no significant differences between the two groups (all *p*s > .2); both groups also did not differ in their baseline Elevator Task with Reversal performance (4 hours or fewer = 55.56, 5 hours or more = 55, *p* = .969).

Secondly, we examined the difference in the rate of improvement on the Elevator Task with Reversal between Session 1 and Session 3 in both groups. Those who practiced 5 hours or more performed significantly better on Session 3 than Session 1 (18.75, *t* = 8.275, df = 7, *p* < .001, two-tailed), whereas the improvement in those who practiced 4 hours or fewer was not significant (4.44, *t* = .555, df = 8, *p* = .549, two-tailed) (Table 3).

## 4. Discussion

Our results demonstrate a significant improvement in an attentional switching task (the Elevator Task with Reversal) after a one-week intensive Gaelic course. By comparison, the passive control group did not show any improvement, while the active control group took an intermediate position. These findings expand on the results of previous research [14] by demonstrating for the first time a language learning-related attentional improvement longitudinally *within the same participants*. The improvement was noted across all age groups, from 18 to 78 years old. Although overall the baseline performance on the Elevator Task with Reversal decreased with age, all three age groups demonstrated an improved score after the language course. Previous research shows that aging does not equally affect all aspects of language [18]; our results suggest that it does not diminish the cognitive effects of language learning. Moreover, the improvement did not depend on Gaelic knowledge–in fact, the less advanced groups displayed a larger effect than the more advanced one. It would be tempting to assume that such pronounced effects after only one week of language learning would be short-lived. However, the improvement persisted in all participants who practiced 5 hours of Gaelic or more per week.

Much recent research on bilingualism focuses on the ease with which highly proficient, balanced, early bilinguals navigate between their languages in everyday life [19]. In contrast, our approach investigates adult language learners in the early stages of language mastery, stressing the role of effort and practice [20] and linking it to the emerging literature on cognitive reserve [21], which postulates that mental exercise (including bilingualism) can compensate to a certain degree for the effects of cognitive aging [6] as well as for pathological brain processes such as dementia [3] or stroke [22].

Cognitive training has been shown to lead to measurable improvements beyond the practiced task, with gains independent of the age of participants or total training time [23–24]. Specifically, “*novel*, *cognitively challenging activities*” seem to be more effective in improving performance on tests of cognitive functions, such as working memory, than less taxing, familiar ones [15]. This could also apply to language learning and use. In this respect, our study corroborates previous findings showing differences in electrophysiological responses to executive tasks after 6 months of an introductory Spanish course [9] and an improvement on executive control tasks after a 20-day training program in conversational French in 4–6 year old children [17].

Interestingly, the study by Janus et al [17] also found improvements on executive tasks in a group undergoing musical instruction of the same duration. It is important to emphasize that although our study focused primarily on language learning, a positive effect on attention switching was also observed in the active control group, which was engaged in intensive courses not related to language learning. Likewise, in the follow-up study, a lasting improvement was also seen in some of the participants who practiced 4 hours a week or less: it was just not as consistent as the improvement in those who practiced 5 hours or more. An improvement in cognitive functions can be achieved through a wide range of mental activities [15, 16]; future research will need to examine not only specific types of mental exercise but also their possible combinations and interactions.

The improvement in attention did not depend on the level of previous knowledge of Gaelic and was also detectable in the very beginners. This could point to the importance of the “*desirable difficulties”* [25] of *novelty*, *challenge* and *effort*. Many studies in basic neuroscience stress the importance of novelty for neurogenesis, synaptic tagging, and memory formation [26, 27]. One of the fundamental issues in this context is the interaction between novelty, facilitating the formation of new synaptic connections, and familiarity, supporting their maintenance. Indeed, while novelty could have helped our participants to achieve an improvement in attention, it was the *continuous practice*, which determined whether such changes persisted 9 months after the language course. Interestingly, one of the largest studies of multilingualism in aging [2] found the best cognitive performance in participants who most frequently used a language other than their native tongue. Our study postulates, therefore, the importance of both novelty and practice.

Our study has limitations. The enrolment in the courses determined the number of participants recruited, while the short time in which the testing had to be conducted, at the beginning and end of the course, imposed constraints on the number of tests used. The participants were not randomly assigned and the geographic spread of their domiciles meant that we could only follow-up on half of them. All three groups consisted of people who either enrolled in different educational courses or signed up for the Psychology Volunteer Panel, which includes regular participation in cognitive experiments. Hence, they could be perceived as particularly keen to engage in cognitive activities and not necessarily representative of the overall population (however, the three groups were highly comparable with each other in this respect). Finally, in the analysis of the longitudinal data, we set the threshold of 4 or fewer versus 5 or more hours of practice per week based on the inspection of our results and not on previous theoretical insights, so its relevance will need to be confirmed in future studies.

However, within the limits of the achievable, our results are remarkably clear and consistent. Our groups did not differ with respect to demographic variables and baseline performance. The Elevator Task with Reversal improvement did not depend on age or knowledge of Gaelic; if anything, it was stronger in the beginners. Not a single participant who practiced Gaelic for 5 or more hours/week deteriorated in his/her performance compared to the baseline. Hence, we hope that our work will encourage further research into language learning as a form of cognitive training, drawing attention to the importance of novelty, challenge, and practice.

## Supporting Information

S1 Appendix Language Background Questionnaire. (DOC)

S2 Appendix Data from Original Study. (XLS)

S3 Appendix Follow-up Data. (XLS)
