# Supplementary material for: Novelty, Challenge, and Practice: The Impact of Intensive Language Learning on Attentional Functions
Source: PLoS One. 2016 Apr 27;11(4):e0153485. doi: 10.1371/journal.pone.0153485 (PMC4847793; doi:10.1371/journal.pone.0153485)
Supplement: S1 Appendix — (DOC) [file pone.0153485.s001.doc]

|  | | | | | | | | | | | | | | Participant ID | | | |
| --- | --- | --- | --- | --- | --- | --- | --- | --- | --- | --- | --- | --- | --- | --- | --- | --- | --- |
| **PART I: GENERAL DEMOGRAPHIC INFORMATION** | | | | | | | | | | | | | | | | | |
| Age....................................................................Gender........................................Handedness................  Profession/Subject studied.....................................................................................................................  Current level of study or highest level achieved....................................................................................  At what age did you start school? At ............ yrs old  Have you lived in other places in which other languages are spoken?...................................................  If so, where and for how long?................................................................................................................ .................................................................................................................................................................  Place of birth:......................................................................................................................................... | | | | | | | | | | | | | | | | | |
| **PART II: LANGUAGES SPOKEN** | | | | | | | | | | | | | | | | | |
| 1. | | | | | | | | | | | | | | | | | |
| 2. | | | | | | | | | | | | | | | | | |
| 3. | | | | | | | | | | | | | | | | | |
| 4. | | | | | | | | | | | | | | | | | |
| 5. | | | | | | | | | | | | | | | | | |
| **PART III: PARENTS’ MOTHER TONGUES:** | | | | | | | | | | | | | | | | | |
| What is your father’s mother tongue?  Does your father speak any other languages? Please specify | | | | | | | | | | | | | | | | | |
| What is your mother’s mother tongue?  Does your mother speak any other languages? Please specify | | | | | | | | | | | | | | | | | |
| If you have a child, which is their first language?  Does your child speak any other languages? Please specify | | | | | | | | | | | | | | | | | |
| **LANGUAGE 1:** | | | | | | | | | | | | | | | | | |
| **LANGUAGE HISTORY – ACQUISITION OF LANGUAGE** | | | | | | | | | | | | | | | | | |
| 1. First contact with the language: since birth/at ___ yrs of age | | | | | | | | | | | | | | | | | |
| 2. Choose the most appropriate option: - I hear the language spoken but I don’t speak it 🞎  - I both hear and speak the language 🞎 | | | | | | | | | | | | | | | | | |
| 3. If you were schooled in this language, at what age did you learn to write it? ____ yrs old | | | | | | | | | | | | | | | | | |
| 4. Environment in which you used the language in childhood: Frequency of use (choose one): | | | | | | | | | | | | | | | | | |
|  | **Always** | | | **Often** | | | **Sometimes** | | | | **Rarely** | | | | **Never** | | **Not applicable** |
| ***Family*** | | | | | | | | | | | | | | | | | |
| Mother |  | | |  | | |  | | | |  | | | |  | |  |
| Father |  | | |  | | |  | | | |  | | | |  | |  |
| Grandparents |  | | |  | | |  | | | |  | | | |  | |  |
| Siblings |  | | |  | | |  | | | |  | | | |  | |  |
| Other relatives |  | | |  | | |  | | | |  | | | |  | |  |
| ***Official*** | | | | | | | | | | | | | | | | | |
| Schooling |  | | |  | | |  | | | |  | | | |  | |  |
| Teachers |  | | |  | | |  | | | |  | | | |  | |  |
| Classmates |  | | |  | | |  | | | |  | | | |  | |  |
| ***Immediate Environment*** | | | | | | | | | | | | | | | | | |
| Friends |  | | |  | | |  | | | |  | | | |  | |  |
| Neighbours |  | | |  | | |  | | | |  | | | |  | |  |
|  | | | | | | | | | | | | | | | | | |
| **LANGUAGE USE** | | | | | | | | | | | | | | | | | |
| 1. Do you continue to use the language? Yes🞎 No🞎 (If no, when did you stop using it? ___ yrs old) | | | | | | | | | | | | | | | | | |
| If yes, how often do you use it in each one of the following contexts (choose one) | | | | | | | | | | | | | | | | | |
|  | | | **Always** | | **Often** | | | **Sometimes** | | | | **Rarely** | | | **Never** | **Not applicable** | |
| ***Family*** | | | | | | | | | | | | | | | | | |
| Partner | | |  | |  | | |  | | | |  | | |  |  | |
| Siblings/Nephews/Nieces | | |  | |  | | |  | | | |  | | |  |  | |
| Children | | |  | |  | | |  | | | |  | | |  |  | |
| Other relatives | | |  | |  | | |  | | | |  | | |  |  | |
| ***Official*** | | | | | | | | | | | | | | | | | |
| Colleagues | | |  | |  | | |  | | | |  | | |  |  | |
| Shopping | | |  | |  | | |  | | | |  | | |  |  | |
| Radio/TV | | |  | |  | | |  | | | |  | | |  |  | |
| Books/magazines | | |  | |  | | |  | | | |  | | |  |  | |
| ***Immediate environment*** | | | | | | | | | | | | | | | | | |
| Friends | | |  | |  | | |  | | | |  | | |  |  | |
| Neighbours | | |  | |  | | |  | | | |  | | |  |  | |
| Church/society | | |  | |  | | |  | | | |  | | |  |  | |
| Do you use different languages with the same person? | | | | | | | | | | | | | | | | | |
|  | | |  | |  | | | |  | | | |  | | |  | |
| **COMMAND OF THE LANGUAGE** | | | | | | | | | | | | | | | | | |
| Evaluate your command of the language in each of the following categories: | | | | | | | | | | | | | | | | | |
|  | | **Basic** | | | | **Weak** | | | | **Moderate** | | | **Advanced** | | | **Fluent** | |
| Expression | |  | | | |  | | | |  | | |  | | |  | |
| Comprehension | |  | | | |  | | | |  | | |  | | |  | |
| Reading | |  | | | |  | | | |  | | |  | | |  | |
| Writing | |  | | | |  | | | |  | | |  | | |  | |
| **LANGUAGE 2:** | | | | | | | | | | | | | | | | | |
| **LANGUAGE HISTORY – ACQUISITION OF LANGUAGE** | | | | | | | | | | | | | | | | | |
| 1. First contact with the language: since birth/at ___ yrs of age | | | | | | | | | | | | | | | | | |
| 2. Choose the most appropriate option: - I hear the language spoken but I don’t speak it 🞎  - I both hear and speak the language 🞎 | | | | | | | | | | | | | | | | | |
| 3. If you were schooled in this language, at what age did you learn to write it? ____ yrs old | | | | | | | | | | | | | | | | | |
| 4. Environment in which you used the language in childhood: Frequency of use (choose one): | | | | | | | | | | | | | | | | | |
|  | **Always** | | | **Often** | | | **Sometimes** | | | | **Rarely** | | | | **Never** | | **Not applicable** |
| ***Family*** | | | | | | | | | | | | | | | | | |
| Mother |  | | |  | | |  | | | |  | | | |  | |  |
| Father |  | | |  | | |  | | | |  | | | |  | |  |
| Grandparents |  | | |  | | |  | | | |  | | | |  | |  |
| Siblings |  | | |  | | |  | | | |  | | | |  | |  |
| Other relatives |  | | |  | | |  | | | |  | | | |  | |  |
| ***Official*** | | | | | | | | | | | | | | | | | |
| Schooling |  | | |  | | |  | | | |  | | | |  | |  |
| Teachers |  | | |  | | |  | | | |  | | | |  | |  |
| Classmates |  | | |  | | |  | | | |  | | | |  | |  |
| ***Immediate Environment*** | | | | | | | | | | | | | | | | | |
| Friends |  | | |  | | |  | | | |  | | | |  | |  |
| Neighbours |  | | |  | | |  | | | |  | | | |  | |  |
|  | | | | | | | | | | | | | | | | | |
| **LANGUAGE USE** | | | | | | | | | | | | | | | | | |
| 1. Do you continue to use the language? Yes🞎 No🞎 (If no, when did you stop using it? ___ yrs old) | | | | | | | | | | | | | | | | | |
| If yes, how often do you use it in each one of the following contexts (choose one) | | | | | | | | | | | | | | | | | |
|  | | | **Always** | | **Often** | | | **Sometimes** | | | | **Rarely** | | | **Never** | **Not applicable** | |
| ***Family*** | | | | | | | | | | | | | | | | | |
| Partner | | |  | |  | | |  | | | |  | | |  |  | |
| Siblings/Nephews/Nieces | | |  | |  | | |  | | | |  | | |  |  | |
| Children | | |  | |  | | |  | | | |  | | |  |  | |
| Other relatives | | |  | |  | | |  | | | |  | | |  |  | |
| ***Official*** | | | | | | | | | | | | | | | | | |
| Colleagues | | |  | |  | | |  | | | |  | | |  |  | |
| Shopping | | |  | |  | | |  | | | |  | | |  |  | |
| Radio/TV | | |  | |  | | |  | | | |  | | |  |  | |
| Books/magazines | | |  | |  | | |  | | | |  | | |  |  | |
| ***Immediate environment*** | | | | | | | | | | | | | | | | | |
| Friends | | |  | |  | | |  | | | |  | | |  |  | |
| Neighbours | | |  | |  | | |  | | | |  | | |  |  | |
| Church/society | | |  | |  | | |  | | | |  | | |  |  | |
| Do you use different languages with the same person? | | | | | | | | | | | | | | | | | |
|  | | |  | |  | | | |  | | | |  | | |  | |
| **COMMAND OF THE LANGUAGE** | | | | | | | | | | | | | | | | | |
| Evaluate your command of the language in each of the following categories: | | | | | | | | | | | | | | | | | |
|  | | **Basic** | | | | **Weak** | | | | **Moderate** | | | **Advanced** | | | **Fluent** | |
| Expression | |  | | | |  | | | |  | | |  | | |  | |
| Comprehension | |  | | | |  | | | |  | | |  | | |  | |
| Reading | |  | | | |  | | | |  | | |  | | |  | |
| Writing | |  | | | |  | | | |  | | |  | | |  | |
| **LANGUAGE 3:** | | | | | | | | | | | | | | | | | |
| **LANGUAGE HISTORY – ACQUISITION OF LANGUAGE** | | | | | | | | | | | | | | | | | |
| 1. First contact with the language: since birth/at ___ yrs of age | | | | | | | | | | | | | | | | | |
| 2. Choose the most appropriate option: - I hear the language spoken but I don’t speak it 🞎  - I both hear and speak the language 🞎 | | | | | | | | | | | | | | | | | |
| 3. If you were schooled in this language, at what age did you learn to write it? ____ yrs old | | | | | | | | | | | | | | | | | |
| 4. Environment in which you used the language in childhood: Frequency of use (choose one): | | | | | | | | | | | | | | | | | |
|  | **Always** | | | **Often** | | | **Sometimes** | | | | **Rarely** | | | | **Never** | | **Not applicable** |
| ***Family*** | | | | | | | | | | | | | | | | | |
| Mother |  | | |  | | |  | | | |  | | | |  | |  |
| Father |  | | |  | | |  | | | |  | | | |  | |  |
| Grandparents |  | | |  | | |  | | | |  | | | |  | |  |
| Siblings |  | | |  | | |  | | | |  | | | |  | |  |
| Other relatives |  | | |  | | |  | | | |  | | | |  | |  |
| ***Official*** | | | | | | | | | | | | | | | | | |
| Schooling |  | | |  | | |  | | | |  | | | |  | |  |
| Teachers |  | | |  | | |  | | | |  | | | |  | |  |
| Classmates |  | | |  | | |  | | | |  | | | |  | |  |
| ***Immediate Environment*** | | | | | | | | | | | | | | | | | |
| Friends |  | | |  | | |  | | | |  | | | |  | |  |
| Neighbours |  | | |  | | |  | | | |  | | | |  | |  |
|  | | | | | | | | | | | | | | | | | |
| **LANGUAGE USE** | | | | | | | | | | | | | | | | | |
| 1. Do you continue to use the language? Yes🞎 No🞎 (If no, when did you stop using it? ___ yrs old) | | | | | | | | | | | | | | | | | |
| If yes, how often do you use it in each one of the following contexts (choose one) | | | | | | | | | | | | | | | | | |
|  | | | **Always** | | **Often** | | | **Sometimes** | | | | **Rarely** | | | **Never** | **Not applicable** | |
| ***Family*** | | | | | | | | | | | | | | | | | |
| Partner | | |  | |  | | |  | | | |  | | |  |  | |
| Siblings/Nephews/Nieces | | |  | |  | | |  | | | |  | | |  |  | |
| Children | | |  | |  | | |  | | | |  | | |  |  | |
| Other relatives | | |  | |  | | |  | | | |  | | |  |  | |
| ***Official*** | | | | | | | | | | | | | | | | | |
| Colleagues | | |  | |  | | |  | | | |  | | |  |  | |
| Shopping | | |  | |  | | |  | | | |  | | |  |  | |
| Radio/TV | | |  | |  | | |  | | | |  | | |  |  | |
| Books/magazines | | |  | |  | | |  | | | |  | | |  |  | |
| ***Immediate environment*** | | | | | | | | | | | | | | | | | |
| Friends | | |  | |  | | |  | | | |  | | |  |  | |
| Neighbours | | |  | |  | | |  | | | |  | | |  |  | |
| Church/society | | |  | |  | | |  | | | |  | | |  |  | |
| Do you use different languages with the same person? | | | | | | | | | | | | | | | | | |
|  | | |  | |  | | | |  | | | |  | | |  | |
| **COMMAND OF THE LANGUAGE** | | | | | | | | | | | | | | | | | |
| Evaluate your command of the language in each of the following categories: | | | | | | | | | | | | | | | | | |
|  | | **Basic** | | | | **Weak** | | | | **Moderate** | | | **Advanced** | | | **Fluent** | |
| Expression | |  | | | |  | | | |  | | |  | | |  | |
| Comprehension | |  | | | |  | | | |  | | |  | | |  | |
| Reading | |  | | | |  | | | |  | | |  | | |  | |
| Writing | |  | | | |  | | | |  | | |  | | |  | |
| **LANGUAGE 4:** | | | | | | | | | | | | | | | | | |
| **LANGUAGE HISTORY – ACQUISITION OF LANGUAGE** | | | | | | | | | | | | | | | | | |
| 1. First contact with the language: since birth/at ___ yrs of age | | | | | | | | | | | | | | | | | |
| 2. Choose the most appropriate option: - I hear the language spoken but I don’t speak it 🞎  - I both hear and speak the language 🞎 | | | | | | | | | | | | | | | | | |
| 3. If you were schooled in this language, at what age did you learn to write it? ____ yrs old | | | | | | | | | | | | | | | | | |
| 4. Environment in which you used the language in childhood: Frequency of use (choose one): | | | | | | | | | | | | | | | | | |
|  | **Always** | | | **Often** | | | **Sometimes** | | | | **Rarely** | | | | **Never** | | **Not applicable** |
| ***Family*** | | | | | | | | | | | | | | | | | |
| Mother |  | | |  | | |  | | | |  | | | |  | |  |
| Father |  | | |  | | |  | | | |  | | | |  | |  |
| Grandparents |  | | |  | | |  | | | |  | | | |  | |  |
| Siblings |  | | |  | | |  | | | |  | | | |  | |  |
| Other relatives |  | | |  | | |  | | | |  | | | |  | |  |
| ***Official*** | | | | | | | | | | | | | | | | | |
| Schooling |  | | |  | | |  | | | |  | | | |  | |  |
| Teachers |  | | |  | | |  | | | |  | | | |  | |  |
| Classmates |  | | |  | | |  | | | |  | | | |  | |  |
| ***Immediate Environment*** | | | | | | | | | | | | | | | | | |
| Friends |  | | |  | | |  | | | |  | | | |  | |  |
| Neighbours |  | | |  | | |  | | | |  | | | |  | |  |
|  | | | | | | | | | | | | | | | | | |
| **LANGUAGE USE** | | | | | | | | | | | | | | | | | |
| 1. Do you continue to use the language? Yes🞎 No🞎 (If no, when did you stop using it? ___ yrs old) | | | | | | | | | | | | | | | | | |
| If yes, how often do you use it in each one of the following contexts (choose one) | | | | | | | | | | | | | | | | | |
|  | | | **Always** | | **Often** | | | **Sometimes** | | | | **Rarely** | | | **Never** | **Not applicable** | |
| ***Family*** | | | | | | | | | | | | | | | | | |
| Partner | | |  | |  | | |  | | | |  | | |  |  | |
| Siblings/Nephews/Nieces | | |  | |  | | |  | | | |  | | |  |  | |
| Children | | |  | |  | | |  | | | |  | | |  |  | |
| Other relatives | | |  | |  | | |  | | | |  | | |  |  | |
| ***Official*** | | | | | | | | | | | | | | | | | |
| Colleagues | | |  | |  | | |  | | | |  | | |  |  | |
| Shopping | | |  | |  | | |  | | | |  | | |  |  | |
| Radio/TV | | |  | |  | | |  | | | |  | | |  |  | |
| Books/magazines | | |  | |  | | |  | | | |  | | |  |  | |
| ***Immediate environment*** | | | | | | | | | | | | | | | | | |
| Friends | | |  | |  | | |  | | | |  | | |  |  | |
| Neighbours | | |  | |  | | |  | | | |  | | |  |  | |
| Church/society | | |  | |  | | |  | | | |  | | |  |  | |
| Do you use different languages with the same person? | | | | | | | | | | | | | | | | | |
|  | | |  | |  | | | |  | | | |  | | |  | |
| **COMMAND OF THE LANGUAGE** | | | | | | | | | | | | | | | | | |
| Evaluate your command of the language in each of the following categories: | | | | | | | | | | | | | | | | | |
|  | | **Basic** | | | | **Weak** | | | | **Moderate** | | | **Advanced** | | | **Fluent** | |
| Expression | |  | | | |  | | | |  | | |  | | |  | |
| Comprehension | |  | | | |  | | | |  | | |  | | |  | |
| Reading | |  | | | |  | | | |  | | |  | | |  | |
| Writing | |  | | | |  | | | |  | | |  | | |  | |
| **LANGUAGE 5:** | | | | | | | | | | | | | | | | | |
| **LANGUAGE HISTORY – ACQUISITION OF LANGUAGE** | | | | | | | | | | | | | | | | | |
| 1. First contact with the language: since birth/at ___ yrs of age | | | | | | | | | | | | | | | | | |
| 2. Choose the most appropriate option: - I hear the language spoken but I don’t speak it 🞎  - I both hear and speak the language 🞎 | | | | | | | | | | | | | | | | | |
| 3. If you were schooled in this language, at what age did you learn to write it? ____ yrs old | | | | | | | | | | | | | | | | | |
| 4. Environment in which you used the language in childhood: Frequency of use (choose one): | | | | | | | | | | | | | | | | | |
|  | **Always** | | | **Often** | | | **Sometimes** | | | | **Rarely** | | | | **Never** | | **Not applicable** |
| ***Family*** | | | | | | | | | | | | | | | | | |
| Mother |  | | |  | | |  | | | |  | | | |  | |  |
| Father |  | | |  | | |  | | | |  | | | |  | |  |
| Grandparents |  | | |  | | |  | | | |  | | | |  | |  |
| Siblings |  | | |  | | |  | | | |  | | | |  | |  |
| Other relatives |  | | |  | | |  | | | |  | | | |  | |  |
| ***Official*** | | | | | | | | | | | | | | | | | |
| Schooling |  | | |  | | |  | | | |  | | | |  | |  |
| Teachers |  | | |  | | |  | | | |  | | | |  | |  |
| Classmates |  | | |  | | |  | | | |  | | | |  | |  |
| ***Immediate Environment*** | | | | | | | | | | | | | | | | | |
| Friends |  | | |  | | |  | | | |  | | | |  | |  |
| Neighbours |  | | |  | | |  | | | |  | | | |  | |  |
|  | | | | | | | | | | | | | | | | | |
| **LANGUAGE USE** | | | | | | | | | | | | | | | | | |
| 1. Do you continue to use the language? Yes🞎 No🞎 (If no, when did you stop using it? ___ yrs old) | | | | | | | | | | | | | | | | | |
| If yes, how often do you use it in each one of the following contexts (choose one) | | | | | | | | | | | | | | | | | |
|  | | | **Always** | | **Often** | | | **Sometimes** | | | | **Rarely** | | | **Never** | **Not applicable** | |
| ***Family*** | | | | | | | | | | | | | | | | | |
| Partner | | |  | |  | | |  | | | |  | | |  |  | |
| Siblings/Nephews/Nieces | | |  | |  | | |  | | | |  | | |  |  | |
| Children | | |  | |  | | |  | | | |  | | |  |  | |
| Other relatives | | |  | |  | | |  | | | |  | | |  |  | |
| ***Official*** | | | | | | | | | | | | | | | | | |
| Colleagues | | |  | |  | | |  | | | |  | | |  |  | |
| Shopping | | |  | |  | | |  | | | |  | | |  |  | |
| Radio/TV | | |  | |  | | |  | | | |  | | |  |  | |
| Books/magazines | | |  | |  | | |  | | | |  | | |  |  | |
| ***Immediate environment*** | | | | | | | | | | | | | | | | | |
| Friends | | |  | |  | | |  | | | |  | | |  |  | |
| Neighbours | | |  | |  | | |  | | | |  | | |  |  | |
| Church/society | | |  | |  | | |  | | | |  | | |  |  | |
| Do you use different languages with the same person? | | | | | | | | | | | | | | | | | |
|  | | |  | |  | | | |  | | | |  | | |  | |
| **COMMAND OF THE LANGUAGE** | | | | | | | | | | | | | | | | | |
| Evaluate your command of the language in each of the following categories: | | | | | | | | | | | | | | | | | |
|  | | **Basic** | | | | **Weak** | | | | **Moderate** | | | **Advanced** | | | **Fluent** | |
| Expression | |  | | | |  | | | |  | | |  | | |  | |
| Comprehension | |  | | | |  | | | |  | | |  | | |  | |
| Reading | |  | | | |  | | | |  | | |  | | |  | |
| Writing | |  | | | |  | | | |  | | |  | | |  | |

Would you be happy to be contacted again to participate in other experiments? Yes / No

If yes, please provide your contact information: ________________________________________________________________________
